# Comparing clomiphen citrate plus HMG with clomiphen citrate plus rFSH in IUI cycles in couples with unexplained or male factor infertility: A prospective randomized study

**Published:** 2013-03

**Authors:** Azam Azargoon, Marjan Bahrami, Jafar Alavy Toussy

**Affiliations:** *Department of Infertility, Amir-AL-Momenin Hospital, Semnan University of Medical Sciences, Semnan, Iran.*

**Keywords:** *Clomiphen citrate*, *Gonadotropins*, *Intra uterine insemination*, *Ovulation induction*

## Abstract

**Background: **Different protocols are used for controlled ovarian hyper stimulation (COH), but the optimal method has not yet been determined.

**Objective:** The aim of this study was to compare the outcome of controlled ovarian stimulation (COS) using clomiphen citrate (CC) plus HMG versus CC plus rFSH in intra uterine insemination cycles (IUI).

**Materials and Methods:** 144 women with unexplained or male factor infertility undergoing IUI cycles were randomized (72 patients in CC plus rFSH group and 72 patients in CC plus HMG group) and included in this single blind study from October 2006 to June 2010. The primary outcomes were clinical and ongoing pregnancy rates. The number of dominant follicles, mean of follicular size, endometrial thickness on the day of HCG administration, total dose of gonadotropins and duration of stimulation with gonadotropins were secondary outcomes.

**Results: **Clinical and ongoing pregnancy rates were not significantly different in the two groups .There was a significant higher multiple pregnancy rate in CC plus rFSH group (33.3%) versus CC plus HMG group (12.5%; p<0.005). There were no statistically significant differences in the secondary outcomes between the two groups.

**Conclusion: **According to our results it seems that CC plus HMG is a more suitable and cost-effective regimen than CC plus rFSH in IUI cycles in patients with unexplained or male factor infertility.

## Introduction

Homologous intra uterine insemination (IUI) preceded by controlled ovarian hyperstimulation (COH) is a less expensive and invasive treatment than other assisted reproductive techniques. It is the best first line treatment and the most effective procedure for unexplained and male factor subfertility (1). Several studies suggest that IUI cycles with ovarian stimulation improves the probability of conception in couples with unexplained infertility but the main concern is the efficacy of the agent which has been used for stimulation (2-7). Hyper stimulation with clomiphen citrate (CC) was shown to be the cost-effective treatment for unexplained infertility, although the use of gonadotropin seemed to be a more efficatious option in IUI treatment (8).

Although several drugs with different hormone content and characteristics were available, there was no clear evidence for a superior effect according to the type of gonadotropine used in IUI cycles (9). During the past decade the production of rFSH has been considered as a milestone in the endocrine research, particularly in the field of human reproduction. The urinary preparations of gonadotropins have been progressively replaced by recombinant products which have many advantages including; independency from urine collection, ensuring a constant supply and guaranteeing batch-to-batch consistency but they had higher medical costs (10). A few investigations have prospectively compared recombinant FSH with HMG or uFSH with or without GnRH-agonist down regulation for ovarian stimulation in IUI cycles (10-13).

On the other hand, the choice of using gonadotropin is authority- based, and the best gonadotropin regimen that achieves the highest pregnancy rates in IUI cycles is also controversial (11). Considering the fact that controlled ovarian stimulation (COS) with CC plus HMG is routinely used in many infertility centres in Iran for ovarian stimulation in IUI cycles, while recombinant FSH is currently considered by many investigators as superior to human derived gonadotropins; we decided to do the first comparative study of the outcomes of CC plus rFSH protocol versus CC plus HMG protocol among a group of Iranian patients with unexplained or male factor infertility in IUI cycles.

## Materials and methods

**Figure 1:**
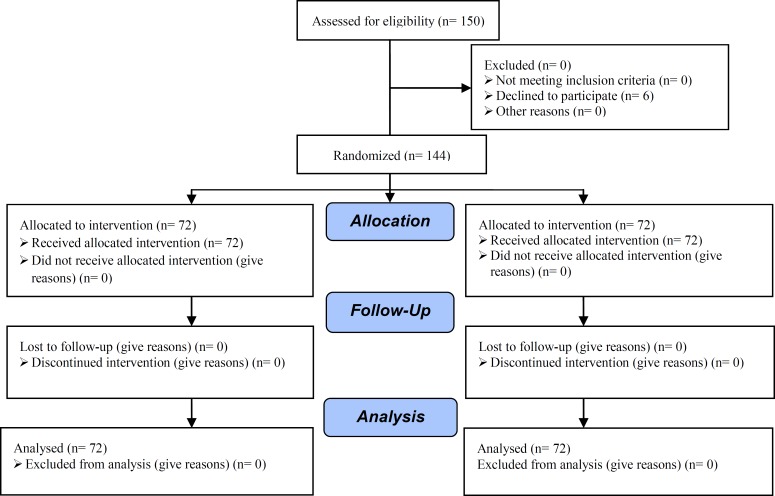
Consort flow diagram

One hundred fifty women were eligible but 6 women refused to participate in the study, so we recruited 144 women with unexplained or male factor infertility undergoing IUI cycles. The selected patients allocated to case and control groups by using systematic randomization. The first patient selected as case by chance using EpiInfo software (WHO and CDC, version 6.4) and the second one to the control group and this approach continued for the rest of patients. Finally 72 patients were allocated to group 1 (CC plus rFSH group) and 72 patients to group 2 (CC plus HMG group) (figure1). The study designed as a single blind clinical trial (the research personels were not informed of the study-group assignment). The study was performed in Amir-Al-Momenin university hospital from October 2006 to June 2010. Ethical committee approval was obtained from the Research Ethics Committee of Semnan University of Medical Sciences before starting the research. All patients provided written informed consent after the methods were fully explained by a nurse. 

Patients were included in the study if they satisfied the following criteria: history of infertility of more than 1 year, women’s age between 20 and 40 years old with their first IUI cycle at the time of study, documentation of normal ovulatory cycles, normal TSH and prolactin level, normal reproductive hormones in the early follicular phase, having patent tubes shown by HSG or laparoscopy and having a normal sperm count, motility and morphology according to the World Health Organization (WHO 1992) criteria for unexplained infertility and the total motile sperm count being more than 1 million in male factor infertility. 

Patients with a history of renal or liver failure, cardiovascular disease and diabetes were excluded. Patients were then randomly assigned to two groups; group 1 received CC plus rFSH (Gonal F, Serono, Turkey; costing $17 per 75 IU ampoule) and group 2 received CC plus HMG (Merional, HP, Serono, Turkey; costing $10 per 75 IU ampoule). Ovarian cycle stimulation was started on the 3^rd^ day of menstruation after the basal transvaginal ultrasound (TV-US) with 100 mg CC for 5 consecutive days. The gonadotropin regimens adopted in each group were rFSH 75 IU for group 1, subcutaneous, daily and HMG 75 IU for group 2, intramuscular, daily from day 7 of menstrual cycle (150 IU for woman≥35 years old).

TV-US was started from day 9 of menstrual cycle every other day for follicular size tracing. The gonadotropin dose was continued until the occurrence of at least one dominant follicle (ovarian follicule ≥16 mm in diameter), then HCG (Profasi, Serono, Turkey) 10'000 IU was administered intramuscularly to trigger final follicular maturation and IUI with a sperm swim-up procedure was performed 35-36 hours later. For the prevention of ovarian hyperstimulation syndrome (OHSS), ovulation was induced by busereline acetate (0.5 mg subcutaneously) if there were ≥5 follicles with a diameter of ≥16 mm on the day of HCG administration. The luteal phase was supported by 400 mg of vaginal progesterone suppository (Cyclogest, Actavis, UK) administered twice a day from the day of IUI for two weeks. 

A blood sample for beta human chorionic gonadotropin (βhCG) was obtained two weeks after IUI for pregnancy confirmation. A clinical pregnancy was determined by the visualization of an embryo with cardiac activity at 7 weeks of gestation in TV-US. In case of pregnancy progesterone was continued till 12 weeks of gestation. The primary outcome measures were clinical and ongoing pregnancy rates. The total rFSH and HMG doses, the number of treatment days, the number of mature follicles, the mean size of mature follicles and the endometrial thickness on the day of HCG administration were secondary outcomes.

**Statistical analysis**

We used PASW (Version 18, © IBM SPSS Inc.) for entering data, calculating descriptive results and performing statistical tests. The association between intervention (treatment groups) and the outcome (clinical and ongoing pregnancy rates) was assessed by Chi-square test and if necessary by Fisher Exact test (Fisher Exact test used when at least one of the cells had less than 5 cases). 

The difference between quantitative variables and probable confounding variables was assessed by T-test and if necessary by nonparametric equivalents (Nonparametric test Mann-Whitney used when the distribution of variable was not normal. We used Kolmogorov-Smirnov test for evaluation of normality). EpiInfo (WHO and CDC, version 6.4) was used for randomization of treatment groups. This study was a pilot study and the sample size used was according to the patients accessible in the study period. In all statistical methods α=0.05 was considered as significant.

## Results

A total of 144 patients were randomized. The two groups of patients were comparable with respect to their demographic and fertility backgrounds. Male factor infertility was more frequent than unexplained infertility in both groups and more than 70% of patients had primary infertility. Mean duration of infertility was approximately 5 years in both groups (Table I). 

In our study total clinical pregnancy and ongoing pregnancy rates per cycle with COS and IUI were 11.8% and 11.1%, respectively. There were no significant differences in clinical and ongoing pregnancy rates between the two groups. (12.5% and12.5% in group 1; 11.1% and 9.7% in group 2, respectively (p=0.998, p=0.787).

Four multiple gestations (two twin and one quadruplet pregnancies in group 1 and one twin pregnancy in group 2) were seen. There was a significant higher multiple pregnancy rate in group 1 (33.3%) versus group 2 (12.5%) (p=0.005).

With regard to the ovarian cycle parameters, there were no significant differences in terms of the duration of stimulation (p=0.295), the total dose of gonadotropins (p=0.332), the number of dominant follicles (p=0.261), the mean follicular size (p=0.805), and the endometrial thickness on the day of HCG administration (p=0.435) and the rate of GnRH agonist used instead of HCG administration for ovulation induction between the two groups (p=0.801) (Table II). 

In three cases of twin pregnancies the number of dominant follicles on the day of HCG administration were 3 and in the quadruplet pregnancy there were 4 dominant follicles. There was one singleton pregnancy in patients for whom busereline acetate (0.5 mg subcutaneously) was administered instead of HCG for the induction of ovulation. In our study one case of moderate OHSS was seen in group 1.

**Table:** Main demographic and infertility baseline characteristics in two groups

**Parameter**	**Group** **1 (CC+rFSH)**	**Group** **2 (CC+HMG)**	** p-value**
Age (Years)	28.7±5.5	27.9±4.5	0.341^a^
BMI (Kg/m^2^) *	25.59±3.4	26.29±4.5	0.397 ^a^
Duration of infertility* (Years)	5.09±2.8	5.38±2.9	0.657 ^a^
Type of infertility			
Primary	73.6%	72.2%	0.998 ^b^
Secondary	26.4%	27.8%	0.998 ^b^
Cause of infertility			
Unexplained infertility	29.2%	37.5%	0.379^ b^
Mild male factor infertility	70.80%	62.5%	0379^ b^

Note: All values are mean±SD except the type and cause of infertility.

^a^ t-test.

^b^ Chi-square test .

**Table II:** Cycle characteristics of patients when receiving either CC+rFSH or CC+HMG for COS in IUI cycles

**Parameters**	**Group 1 (CC+rFSH)**	**Group 2 (CC+HMG** **(**	**P value**
Duration of gonadotropin consumption (days)	4.64±1.2	4.67±1.2	0.295^ a^
Gonadotropin dos (ampoules/cycle)	4.9±1.9	5.2±1.8	0.332^ a^
On the day of HCG administration			
Number of dominant follicles	2.93±1.6	3.25±1.8	0.261^ a^
Follicular size (mm)	17.02±2.07	16.93±2.3	0.805^ a^
Endometrial thickness (mm)	7.29±1.3	7.1±1.6	0.435^ a^
Rate of GnRH-a used	12.5%	12.5%	0.801 ^b^
Clinical pregnancy rate/cycle	12.5%	11.1%	0.998 ^b^
Ongoing pregnancy rate/cycle	12.5%^ a^	9.7%	0.787 ^b^
Multiple pregnancy rate/preg	33.3%	12.5 %	0.005 ^b^

Note: All values are mean±SD except ratethes.

^a^ t-test.

## Discussion

In this study the overall clinical pregnancy rate per cycle with COS and IUI was found to be 11.8% which is comparable to the pregnancy rate in different studies which ranges from 8.6 to 16.7% (9, 14-16). To the best of our knowledge, there has been no study to compare CC plus hMG vs. CC plus rFSH in IUI cycles, but there have been a few studies which compared rFSH with uFSH or hMG in infertile patients in IUI cycles. In Filicori's study, there was no significant difference in clinical pregnancy rate between the patients who used rFSH (17%) and hMG (28%) in IUI cycles (12). Gerli *et al* did not also demonstrate any significant difference in clinical pregnancy rate between rFSH (12.7%) and hMG (11.9%) (10). 

Recently Sagnella *et al* in a prospective randomized study showed that HP-hMG is not inferior compared with rFSH regarding clinical PR (19.7% vs. 21.4%) in couples with unexplained infertility and/or mild-moderate male factor in intrauterine insemination cycles (17). But contrary to these studies Demrol *et al* demonstrated a significantly higher clinical pregnancy rate in rFSH group than uFSH and hMG group (25.9% vs. 13.8% and 12.5%, respectively) (11).

Recently O'Leary *et al* indicated that clinical pregnancy rate in women stimulated with urinary gonadotropins was significantly higher than those stimulated with r-FSH, 22.22% vs. 10.91%. This is related to the qualitative assessment of perifollicular blood flow recorded in the lead follicle on the day of IUI treatment, so in controlled ovarian stimulation and IUI in women who were down regulated, the addition of exogenous LH activity increased the perifollicular blood flow and the potential for clinical pregnancy (13). Clinical pregnancy with different ovarian stimulation protocols (CC+hMG and hMG or CC alone) were compared in some studies.

Mahani concluded that pregnancy rate is higher with hMG alone than with CC or CC plus hMG in IUI cycles, but Rashidi and Sikandar in two seperate studies did not find a significant difference in clinical pregnancy rates between CC or CC plus hMG in IUI cycles. Pregnancy rates with CC plus HMG in these three studies were 7%, 12.12% and 9.6%, respectively (18-20). Although we used 75 units of gonadotropin in our protocol with a combination of CC, our pregnancy rate was comparable with those studies that used 150 units of gonadotropins (18-20). In this study there were no statistically significant differences in any of the secondary outcome measures between the two groups (Table II). Duration and dose of gonadotropin consumption were similar in both groups the same as those in Platteau's study and were opposite to other studies (11-13, 21).

In the Demrol's study the total dose of gonadotropin per cycle was lower in rFSH than hMG group but the duration of treatment with gonadotropin was similar in the two groups of rFSH and hMG (11). In Filicori's study the dose and duration of gonadotropin consumption was lower in hMG group than rFSH group and finally O'Leary *et al* demonstrated that the duration of treatment with rFSH was lower than HMG but the total dose of gonadotropin was similar in both groups (12, 13). 

Total dose and duration of gonadotropin administration in our study was lower than other studies. This advantage may be due to the use of a combination of clomiphen citrate and gonadotropines in our study. The number of dominant follicles on the day of HCG administration was similar in both groups, the same as in two separate studies carried out by Filicori *et al* and Platteau *et al* (12, 21). But in Demrol's study the number of dominant follicles was higher in rFSH group than HMG group (11).

Iatrogenic multiple gestation is one of the most serious risk factors associated with COS and IUI (19). In this study, there was a higher multiple pregnancy rates in CC plus rFSH group (33.3%) versus CC plus HMG group (12.5%). This result was similar to the result of Sagnella *et al* (17). In Demerol’s study multiple pregnancy rate was 10% with rFSH but in Filicori's study the rate of multiple pregnancy was high (5 out of 11 pregnancies; 2 in rFSH and 3 in HMG group) although the difference between the two groups was not significant. On the contrary some studies did not report any multiple pregnancies with HMG alone or rFSH versus uFSH in IUI cycles (10, 18). In this study we observed one high-order multiple pregnancies (one quadruplet pregnancy) in the presence of 4 follicles on the day of HCG administration. We did not cancel any cycle but in patients with ≥5 follicle ≥16mm on the day of HCG administration, busereline acetate was administered instead of HCG.

In other studies the cycle was cancelled when ≥4 dominant follicles or ≥3 dominant follicles were seen on the day of HCG administration (11, 21). The percentage of multiple pregnancies in ovulation induction cycles should be decreased to single digits, and prefered to be <5% as there are substantial social, economic and health consequences of multiple pregnancy (22, 23). So for reducing the chance of multiple pregnancy it is better to cancel the cycles in the presence of ≥4 follicles of ≥16mm on the day of HCG administration.

In our study a case of moderate OHSS happened in CC plus rFSH group, but this rate was not significantly different in the two groups the same as Platteau 's study (21). But recently Sagnella *et al* showed that the number of interrupted cycles for OHSS risk was significantly higher in the rFSH group than in the HP-hMG group (17). The OHSS was not reported in other studies (10-12, 18-20). Considering the remarkable difference in treatment cost between CC plus rFSH and CC plus HMG protocols, the statistically similar pregnancy rate in the two groups, and higher multiple pregnancy rate in CC plus r-FSH protocole than CC plus HMG protocole, it sounds that CC plus HMG protocol is more suitable and cost-effective than CC plus rFSH protocol in COS and IUI cycles in patients with unexplained and male factor infertility. The limited size in both groups is the limitation of our study. So further larger studies are necessary to confirm our results.
